# Osteoprotegerin Is Essential for the Development of Endothelial Dysfunction Induced by Angiotensin II in Mice

**DOI:** 10.3390/ijms25126434

**Published:** 2024-06-11

**Authors:** Mieczysław Dutka, Wojciech Garczorz, Agnieszka Kosowska, Elzbieta Buczek, Piotr Godek, Wojciech Wojakowski, Tomasz Francuz

**Affiliations:** 1 Department of Biochemistry and Molecular Biology, Faculty of Health Sciences, University of Bielsko-Biala, 43-309 Bielsko-Biala, Poland; 2 Department of Biochemistry, Faculty of Medical Sciences in Katowice, Medical University of Silesia, 40-055 Katowice, Poland; wojtekg@sum.edu.pl (W.G.); akosowska@sum.edu.pl (A.K.); tfrancuz@sum.edu.pl (T.F.); 3 Jagiellonian Centre for Experimental Therapeutics (JCET), Jagiellonian University, 30-348 Krakow, Poland; elzbieta.buczek@jcet.eu; 4 Department of Cardiology and Structural Heart Disease, Faculty of Medical Sciences in Katowice, Medical University of Silesia, 40-055 Katowice, Poland; piotrgodek77@gmail.com (P.G.); wwojakowski@sum.edu.pl (W.W.)

**Keywords:** osteoprotegerin, endothelium, endothelial dysfunction, angiotensin II, cytokines, inflammation

## Abstract

Opinions on the effects of osteoprotegerin (OPG) have evolved over the years from a protein protecting the vasculature from calcification to a cardiovascular risk factor contributing to inflammation within the vascular wall. Nowadays, the link between OPG and angiotensin II (Ang II) appears to be particularly important. In this study, the endothelial function was investigated in OPG-knockout mice (B6.129.S4-OPG, OPG^−^) and wild-type (C57BL/6J, OPG^+^) mice under basic conditions and after Ang II exposure by assessing the endothelium-dependent diastolic response of aortic rings to acetylcholine in vitro. A further aim of the study was to compare the effect of Ang II on the expression of cytokines in the aortic wall of both groups of mice. Our study shows that rings from OPG^−^ mice had their normal endothelial function preserved after incubation with Ang II, whereas those from OPG^+^ mice showed significant endothelial dysfunction. We conclude that the absence of OPG, although associated with a pro-inflammatory cytokine profile in the vascular wall, simultaneously protects against Ang II-induced increases in pro-inflammatory cytokines in the murine vascular wall. Our study also demonstrates that the absence of OPG can result in a decrease in the concentration of pro-inflammatory cytokines in the vascular wall after Ang II exposure. The presence of OPG is therefore crucial for the development of Ang II-induced inflammation in the vascular wall and for the development of Ang II-induced endothelial dysfunction.

## 1. Introduction

Osteoprotegerin (OPG) is a glycoprotein that belongs to the tumour necrosis factor superfamily (TNFSF). This superfamily currently comprises more than 20 different ligands and more than 30 related receptors [1]. OPG belongs to the group of soluble receptors and exists mainly in a free form, not bound to the cell membrane [1,2,3]. OPG is best known as a regulator of bone remodelling under physiological conditions and in various clinical situations. The effect of OPG on bone metabolism is mediated by its function as a decoy receptor, which blocks the interaction between the receptor activator of nuclear factor kappa B (RANK) and its ligand (RANKL) [3,4,5,6]. Sites of OPG production include various organs such as kidneys, intestines, stomach and bones, as well as matrix cells, megakaryocytes, immune cells such as B lymphocytes or dendritic cells, vascular endothelial cells (ECs) and vascular smooth muscle cells (VSMCs) [5,7].

A growing body of research confirms that OPG may also be involved in cardiovascular disease processes or may be a prognostic indicator of cardiovascular diseases [6,8]. Initial observations in mice with a knockout of the OPG gene showed that, in addition to increased osteoporosis, there was also increased calcification of the aortic wall and renal arteries in these OPG-deficient animals [7,9,10,11]. 

These first observations formed the basis for viewing OPG as a factor protecting the vasculature from calcification. The similarities between the regulation of osteoclastogenesis in bone and this calcification of the vascular wall and the involvement of the RANK-RANKL-OPG signalling axis in this regulation seemed to support such a hypothesis.

However, subsequent clinical observations have yielded opposite results. High OPG concentrations have been found to be associated with increased cardiovascular risk in patients with coronary artery disease (CAD) [3,5,7,12,13,14]. These high OPG concentrations have also been confirmed as a predictor of a higher incidence of hospitalisation for exacerbation of heart failure (HF) with reduced ejection fraction of ischaemic aetiology [5]. Significant changes in plasma OPG concentrations have also been observed in patients with unstable angina (UA) and in patients with acute myocardial infarction (AMI) [13,15,16,17,18]. Studies have also confirmed that high plasma OPG levels are a strong predictor of cardiovascular death in patients with myocardial infarction, both STEMI (ST-segment elevation myocardial infarction) and NSTEMI (non-ST-segment elevation myocardial infarction) [15,19].

Higher concentrations of OPG have also been found in patients with retinopathy and diabetic neuropathy and in patients with arterial hypertension [5,8]. Therefore, an increased concentration of OPG is regarded as a marker of vascular pathologies associated with diabetes and arterial hypertension, as well as an indicator of endothelial dysfunction and high cardiovascular risk [5,8,12,20]. This has also been confirmed in hypertensive paediatric patients [21]. High plasma OPG concentrations are an indicator of the risk of development of chronic kidney disease (CKD) in patients with arterial hypertension [22]. The detailed mechanisms linking OPG with the regulation of blood pressure are unknown. However, some polymorphisms of the OPG gene, such as rs2073618, have been reported to be associated with the development of hypertension and with a higher cardiovascular risk in patients with type-2 diabetes [23].

The mechanisms of action of OPG that may explain its pathogenic role in arterial wall damage and atherosclerotic plaque development remain unclear. Among other things, OPG is thought to contribute to inflammation within the vascular wall and to enhance leukocyte adhesion to the vascular endothelium [6,24,25]. The importance of angiotensin II (Ang II), platelet-derived growth factor (PDGF), and basic fibroblast growth factor (bFGF) in stimulating OPG expression in VSMCs and contributing to the development of atherosclerotic lesions has been raised [8].

The link between OPG and Ang II, which plays an important role in the development of age-related adverse changes in the phenotype of VSMCs and ECs and in the pathogenesis of atherosclerosis, is of particular relevance [4]. Ang II acts directly on the vascular endothelium, leading to its dysfunction, the development of inflammation, and the progression of atherosclerosis [26]. Reciprocal stimulatory interactions between OPG and Ang II have been demonstrated [3,27,28,29]. Ang II has been shown to increase OPG expression in human and murine vascular walls. OPG has also been shown to increase the expression of the AT1 receptor for Ang II in these cells [3,30]. This is one of the postulated mechanisms by which OPG may promote endothelial dysfunction and the development of atherosclerotic lesions.

Taking into account the links between OPG and Ang II described above and the discrepancies between the results of studies in OPG-knockout mice (suggesting a vascular-protective effect of OPG) and of later preclinical and clinical studies (suggesting an adverse cardiovascular effect of OPG), the aims of our study were established. The first aim of our study was to assess the endothelial function in OPG-knockout mice (OPG^−^) and mice with normal OPG production (OPG^+^). This was achieved by measuring and comparing the acetylocholine-induced endothelium-dependent relaxation response of aortas from OPG^−^ mice and those from OPG^+^ mice, both under basic conditions and after Ang II exposure. A further aim of the study was to assess the effect of Ang II on the expression of individual cytokines in the aortic wall of both these groups of mice. The cytokine profile in the aortic wall of OPG^−^ and OPG^+^ mice was compared. It should be emphasised that the studied mice differed only in the knockout of the OPG gene and in the ability to produce OPG, as the OPG^−^ mice were derived from C57BL/6J wild-type mice (OPG^+^).

## 2. Results

### 2.1. Nitric Oxide-Dependent Endothelial Function in the Isolated Murine Aorta

**Figure 1:**
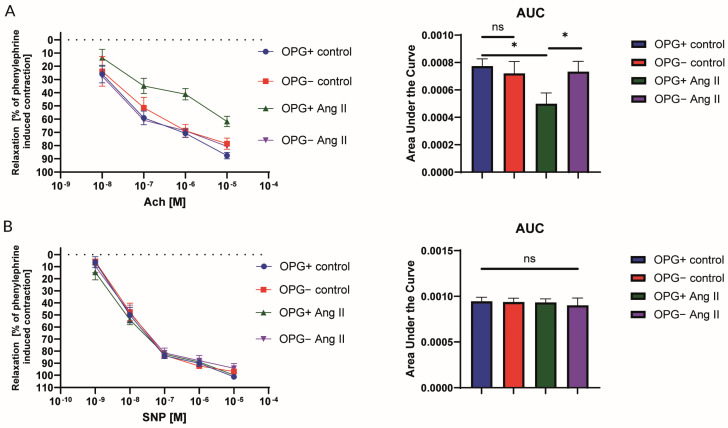
Acetylcholine-induced endothelium-dependent relaxation of aortic rings from wild-type mice (OPG^+^) and OPG-knockout mice (OPG^−^) under basic conditions (control) and after incubation with angiotensin II (Ang II) (**A**). For comparison, sodium nitroprusside-induced endothelium-independent relaxation of aortic rings from OPG^+^ and OPG^−^ mice under basic conditions (control) and after incubation with Ang II is presented (**B**). Mean values ± SEM are shown. n = 9 per group. * *p* < 0.05. Unpaired Student *t*-test.

The endothelial NO-dependent relaxation induced by acetylcholine (Ach) and the endothelium-independent response to sodium nitroprusside (SNP) were tested. In wild-type mice, a significant impairment in the NO-dependent response to ACh was found in the aortic rings incubated for 24 h with Ang II (1 µM) when compared to the control rings (Figure 1A). In contrast, the use of this Ang II-induced endothelial dysfunction model in OPG^−^ mice showed an effect which has not been previously described in the literature. These rings, despite 24 h of incubation with 1 µM of Ang II, retained normal endothelial function and had a normal relaxation response to acetylcholine. As expected, the aortic rings from OPG^+^ mice, incubated for 24 h with Ang II (1 µM), showed a significant impairment in NO-dependent response to ACh when compared to OPG^−^ mice. A comparison of the control rings (not exposed to any agent) from the OPG^−^ and the OPG^+^ mice showed no statistically significant difference in the endothelium-dependent relaxation response to acetylcholine (Figure 1A). There were no differences found in the responses to SNP between all experimental groups (Figure 1B).

### 2.2. Analysis of Cytokine Panel

**Figure 2:**
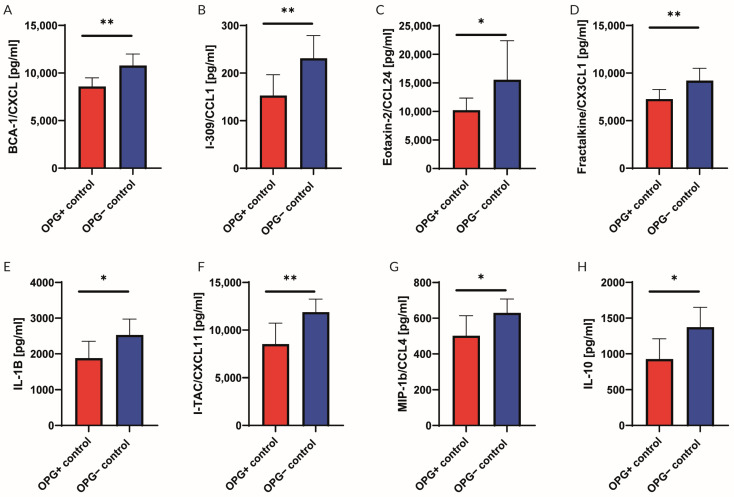
Cytokine concentrations in aortic rings isolated from wild-type mice (OPG^+^) and OPG-knockout mice (OPG^−^). Protein levels of BCA-1/CXCL 13 (**A**), I-309/CCL1 (**B**), Eotaxin-2/CCL24 (**C**), Fractalkine/CX3CL1 (**D**), IL-1B (**E**), I-TAC/CXCL11 (**F**), MIP-1b/CCL2 (**G**) and IL-10 (**H**) were analysed by multiplex. Mean values ± SEM are shown. n = 8 per group. * *p* < 0.05, ** *p* < 0.01. OPG^−^ vs. OPG^+^ under basic conditions (control group). Unpaired Student *t*-test (**A**,**B**,**D**–**H**) and Mann–Whitney test (**C**).

Murine aortic rings isolated from wild-type and OPG-knockout mice were evaluated. Protein levels of BCA-1/CXCL 13, I-309/CCL1, Eotaxin-2/CCL24, Fractalkine/CX3CL1, IL-1B, I-TAC/CXCL11, MIP-1b/CCL2, and IL-10 were analysed via multiplex bead-based (Luminex) assays. The average results from eight murine rings are presented in Figure 2. An increase in pro-inflammatory cytokines was observed in OPG^−^ mice when compared to the OPG^+^ mice. The concentrations of the following pro-inflammatory cytokines increased: Eotaxin-2/CCL24 by 52.0%, BCA-1/CXCL 13 by 25.7%, Fractalkine/CX3CL1 by 26.6%, I-309/CCL1 by 51.0%, IL-1B by 34.4%, I-TAC/CXCL11 by 39.4%, and MCP-1/CCL2 by 18.7%. The anti-inflammatory IL-10 concentration in OPG^−^-knockout mice increased by 48.4% (Figure 2).

**Figure 3:**
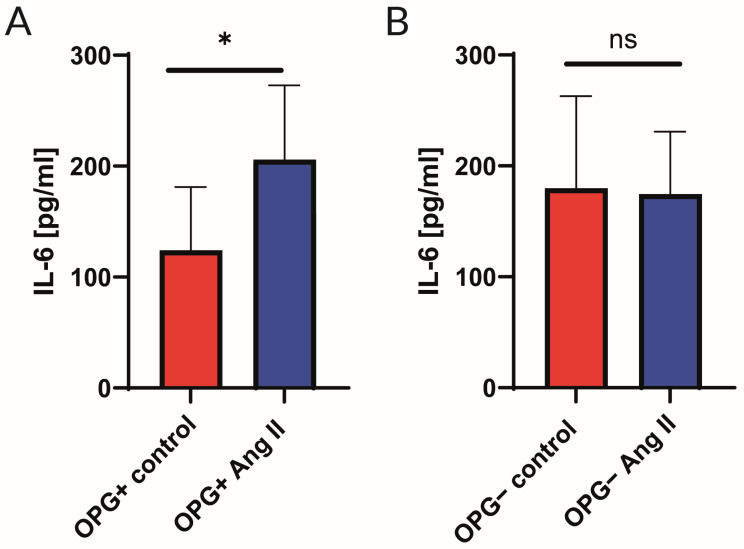
Cytokine concentrations in aortic rings isolated from wild-type mice (OPG^+^) (**A**) and OPG-knockout mice (OPG^−^) (**B**) under basic conditions (control) and after incubation with angiotensin II (Ang II). Protein levels of IL-6 were analysed using multiplex assay. Mean values ± SEM are shown. n = 8 per group. * *p* < 0.05. Mann–Whitney test.

When aortic rings from OPG^+^ and OPG^−^ mice were incubated with 1 µM of Ang II for 24 h and compared with aortic rings incubated in culture medium alone (control), it was found that IL-6 levels in the aortic wall homogenates from OPG^+^ mice increased significantly (77%) after incubation with Ang II. This effect was not observed in the aortic wall of OPG^−^ mice where IL-6 levels remained unchanged despite incubation with Ang II (Figure 3).

**Figure 4:**
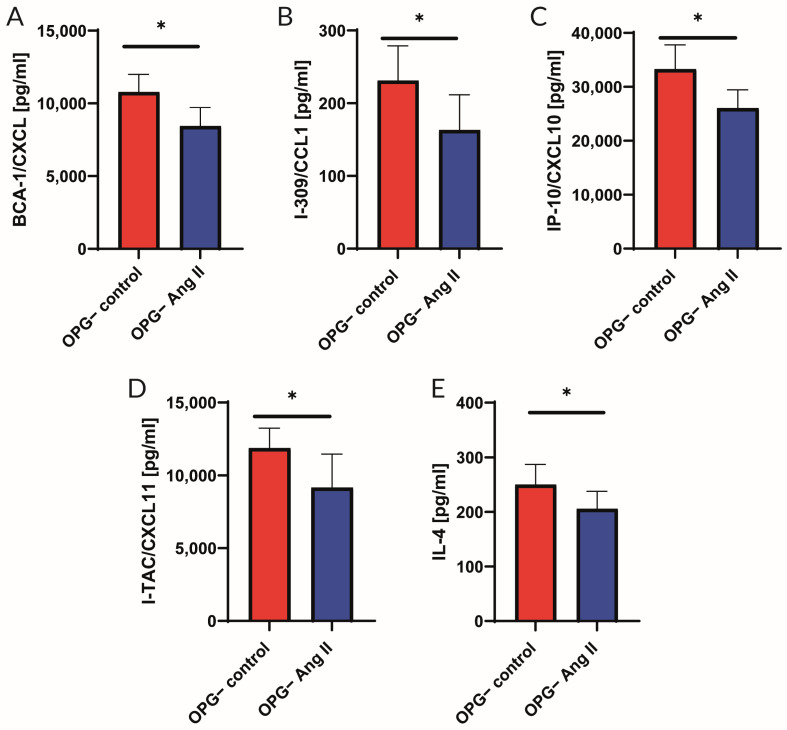
Cytokine concentrations in aortic rings isolated from OPG-knockout mice (OPG^−^) under basic conditions (control) and after incubation with angiotensin II (Ang II). Protein levels of BCA-1/CXCL 13 (**A**), I-309/CCL1 (**B**), IP-10/CXCL 10 (**C**), I-TAC/CXCL11 (**D**), and IL-4 (**E**) were analysed using multiplex assay. Mean values ± SEM are shown. n = 8 per group. * *p* < 0.05. Unpaired Student *t*-test.

Furthermore, OPG^−^ mice showed significantly lower levels of the tested cytokines in aortic ring homogenates incubated with Ang II compared to the control group. No such effect was observed in OPG^+^ mice. In the aortic wall of OPG^−^ mice, the concentrations of the following cytokines decreased upon incubation with Ang II: BCA-1/CXCL 13 by 21.7%, I-309/CCL1 by 29.4%, IL-4 by 17.6%, I-TAC/CXCL11 by 22.8%, and IP-10/CXCL 10 by 21.7% (Figure 4).

## 3. Discussion

### 3.1. Endothelial Function

Given the data indicating the involvement of OPG in endothelial dysfunction and the development of atherosclerotic cardiovascular diseases, the endothelial function was investigated by assessing the endothelium-dependent relaxation response of aortic rings from OPG^+^ and OPG^−^ mice. Due to the reported association between OPG and Ang II, this assessment was performed both under basic conditions and after exposure to Ang II (Figure 1). A comparison of control rings (not exposed to any agent) from OPG^−^ and OPG^+^ mice showed no statistically significant difference in terms of the endothelium-dependent relaxation response to acetylcholine. However, when exposed to Ang II, aortic rings from OPG^−^ mice differed from those from OPG^+^ mice in that they retained normal endothelial function and normal relaxation response to acetylcholine despite 24 h of incubation with Ang II. 

In contrast, aortic rings from OPG^+^ mice showed a significant impairment of the endothelium-dependent relaxation response to acetylcholine after 24 h of incubation with Ang II compared to control rings incubated in the culture medium alone. Such a response is typically observed in a model of Ang II-induced endothelial dysfunction [31]. To our knowledge, this study is the first to show that knockout of the OPG gene protects against Ang II-induced endothelial dysfunction. This suggests a link between the endothelial dysfunction-inducing effects of Ang II and OPG. The OPG^−^ mice used in this experiment are from the same strain as the wild-type (OPG^+^) mice studied. The only difference is that they are unable to produce OPG because the gene for OPG has been knocked out. The presence of OPG is therefore a key factor in the ability of Ang II to induce endothelial dysfunction.

### 3.2. Cytokine Profile

In view of the results obtained in this functional study indicating that OPG is involved in the induction of endothelial dysfunction by Ang II and the well-known link between Ang II action on the vascular wall and inflammation [26], the cytokine profile in the vascular wall of the OPG^+^ and OPG^−^ mice was also assessed. Analysis of cytokines showed that the aortic wall of OPG-knockout mice exhibited a pro-inflammatory profile. The levels of various proteins, including BCA-1/CXCL13, Eotaxin-2/CCL24, Fractalkine/CX3CL1, I-309/CCL1, IL-1B, I-TAC/CXCL11, and MCP-1/CCL2, were significantly increased in the aortic wall of OPG-knockout mice when compared to wild-type mice. These proteins play critical roles in the complex landscape of cardiovascular diseases (CVDs). Each of these proteins contributes to different aspects of the inflammatory and immune responses associated with cardiovascular pathophysiology [32,33,34].

The pro-inflammatory cytokine profile in the aortic wall of OPG^−^ mice found in this study could explain the first observations made by Bucay et al. and Mizuno et al. in mice with a knockout gene for OPG [9,10]. At the time, it was noted that these mice not only had increased osteoporosis and a higher incidence of bone fractures but also had increased calcification of the aortic wall and renal arteries [7,9,10,11]. These early observations provided the basis for considering OPG as a vascular protective factor against calcification, and the similarities between the regulation of osteoclastogenesis in bones and this calcification of the vascular wall and the involvement of the RANK-RANKL-OPG signalling axis in this regulation seemed to support such a hypothesis. However, this was not confirmed by subsequent studies in humans. These studies showed, among other things, that older women with severe osteoporosis have high OPG levels and that these levels are higher the more advanced the osteoporosis [7,35,36]. In addition, high OPG levels in these women correlated strongly and positively with cardiovascular mortality [7,37,38]. Other studies have shown that high OPG concentrations in patients with CAD correlate strongly and positively with the severity of CAD and cardiovascular mortality [3,7,39,40,41]. It has also been confirmed that an increase in OPG levels during acute coronary syndromes is an indicator of the risk of adverse cardiovascular events and poor prognosis [16,17]. This was initially thought to be a protective mechanism, with the belief that OPG levels increase in response to harmful agents and that high OPG levels may have a protective effect or at least be a marker of the potency of the damaging effects of other factors. However, subsequent studies have provided ample evidence of the direct adverse effects of OPG on the blood vessel wall, including increased leukocyte adhesion to the endothelial surface, activation of the renin–angiotensin–system (RAS), pro-inflammatory and pro-fibrotic effects, and the induction of endothelial dysfunction at early stages of atherogenesis [42]. The known mechanisms of OPG action on vascular wall cells now allow a better understanding of the clinical observations, indicating a strong association between high OPG concentrations, the presence and course of CVDs, and the development of HF and cardiovascular mortality. It is now believed that, although, at the initial stage, the increase in OPG concentrations may be a response of the vascular wall to various damaging agents, at a later stage, the persistence of high OPG concentrations results in the activation of mechanisms of adverse OPG action that ultimately contribute to vascular damage and atherogenesis [42]. 

In our study, we also found that incubation of the aortic wall of OPG^+^ mice with Ang II resulted in a significant increase in IL-6 levels (a 77% increase) in the aortic wall under the influence of Ang II. In OPG^−^ mice, this increase in IL-6 was absent in response to Ang II (Figure 3). This indicates a differential response of the vascular wall to Ang II depending on the availability of OPG. This differential response was also observed for other cytokines. Indeed, it was found that in OPG^−^ mice, incubation of aortic rings with Ang II was associated with lower concentrations of the cytokines tested compared to the control group. This was not observed in OPG^+^ mice. The decrease in cytokine levels after incubation with Ang II involved proteins such as BCA-1/CXCL13, I-309/CCL1, IL-4, I-TAC/CXCL11, and IP-10/CXCL10 (Figure 4). Thus, when there was a lack of OPG, there was no Ang II-mediated increase in IL-6 levels, and there was an Ang II-mediated decrease in levels of the aforementioned cytokines. This indicates the importance of the link between OPG and Ang II, which plays a major role in the pathogenesis of atherosclerosis [4]. Previous studies have confirmed that Ang II acts directly on the vascular endothelium, leading to endothelial dysfunction, the development of inflammation, and the progression of atherosclerosis [26]. Reciprocal stimulatory interactions between OPG and Ang II have been demonstrated [3,27,28,29]. Ang II has been shown to increase OPG expression in human and murine vascular cells. OPG has also been shown to increase the expression of the AT1 receptor for Ang II in these cells [3,30]. This is one of the postulated mechanisms by which OPG may promote endothelial dysfunction and the development of atherosclerotic lesions. This may explain the results of our functional myograph studies in which, despite the incubation of the aortic rings with Ang II, a normal endothelium-dependent response to acetylcholine was observed in the absence of OPG in the OPG^−^ mice. The results obtained in both parts of the experiments suggest that the presence of OPG in the model of induction of endothelial dysfunction is crucial for eliciting the inflammatory effect of Ang II.

### 3.3. Mechanisms of Action of OPG in Vascular Wall

There are three ways in which OPG exerts its biological effect on the cells of the vascular walls. Firstly, by attaching itself through a specific domain to its ligand RANKL, OPG prevents RANKL from joining its receptor, RANK. Secondly, OPG triggers cell-surface signalling by direct action on the cells. It achieves this through a heparin-binding domain, which is able to bind to heparan sulphate proteoglycans, which can be found on the surface of cells. Such action has been confirmed in bones as well as in cells of the vascular wall and immune system [3,6,25,43]. Thirdly, OPG diminishes or eliminates the effects of TRAIL. It does this by attaching itself to its ligand TRAIL, thereby preventing TRAIL binding to its receptors [6].

RAS is crucial in the development of adverse changes, which occur with age, in the VSMCs and ECs phenotype, as well as the pathogenesis of atherosclerosis [4]. Direct action on the vascular endothelium by the main mediator of RAS, which is Ang II, causes dysfunction, further inflammation, and the development of atherosclerosis. Heightened expression of vascular endothelial growth factors (VEGFs), VEGF-A and VEGF-B, caused by the activation of the angiotensin II type 1 (AT1) receptor, enhance inflammation and remodelling in blood vessels by triggering pro-inflammatory mechanisms and pathological angiogenesis. These VEGF actions are intensified by OPG [26]. Both atheroma-derived cells harvested during endarterectomy and ECs from human dermal microvasculature were tested under cell culture conditions and cultivated with and without irbesartan, which is the blocker of the AT1 receptor [44]. In these cells, a reduction in the concentration levels of IL-6, interleukin-8 (IL-8), and OPG has been confirmed with the use of irbesartan. Furthermore, the blockade of the AT1 receptor in these cells caused a decrease in extracellular signal-regulated kinase-1 and -2 (ERK1 and ERK2) expression as well as a reduction in their phosphorylation, which is usually activated by RANKL when it is bound to RANK. Similarly, there was also a reduction in RANKL-induced ERK1 and ERK2 phosphorylation in a study with mice using losartan—another blocker of AT1 [45]. This would suggest that, at the level of ERK1/2 phosphorylation regulation, there is a convergent action of RANKL and Ang II. OPG can also directly activate ERK1/2 phosphorylation, which is linked to pathological angiogenesis [4,44,45].

Furthermore, it has been confirmed that mutual stimulating interactions exist between OPG and RAS [3,27,28,29]. Ang II increases OPG expression in vascular wall cells in both humans and mice, while irbesartan reduces the OPG expression in these cells by blocking the AT1 receptor. In the same way that Ang II has a dose-dependent effect in enhancing OPG expression in vascular wall cells, OPG has a dose-dependent effect in enhancing the expression of the AT1 receptor [3,30].

Ang II causes an increase in the expression of VEGF, which results in an over-expression of RANK in ECs and intensifies the angiogenic response of these cells to RANKL. A possible additional RANKL action, which occurs via the PI3-kinase/Akt signalling pathway, is to maintain EC integrity and induce a pro-survival effect on ECs [4]. This effect is inhibited when OPG binds with RANKL, thereby impeding its link with RANK and the activation of the PI3-kinase/Akt signalling pathway. When PI3-kinase is inhibited, the pro-survival effect of RANKL in relation to ECs is blocked [46]. Ang II enhances the expression of VEGF, which suppresses the PI3-kinase/Akt signalling pathway [47]. 

### 3.4. Conclusions, Potential Clinical Implications, and Future Perspectives

Based on our results, we may conclude that the OPG gene knockout protects against Ang II-induced endothelial dysfunction in mice. The absence of OPG, although associated with a pro-inflammatory cytokine profile in the vascular wall, simultaneously protects against Ang II-induced increases in pro-inflammatory cytokine concentrations in the murine vascular wall. As shown in our study, the absence of OPG can even lead to a decrease in the concentration of pro-inflammatory cytokines in the vascular wall exposed to Ang II. The presence of OPG is therefore crucial for the development of Ang II-induced inflammation in the vascular wall and for the development of Ang II-induced endothelial dysfunction. OPG may therefore be a potential therapeutic target in cardiovascular diseases with significant activation of the RAS and consequently high concentrations of Ang II inducing inflammation in the vascular wall and endothelial dysfunction. 

There are several areas of particular interest, one of which is the interaction between OPG and TRAIL, which suppresses the mesenchymal stem cell (MSC) migratory activity caused by TRAIL. This suppressive activity of OPG on MSC migration has been demonstrated in vitro. This is of particular interest because there is, among other things, an increase in MSCs migrating from the bone marrow to the infarcted area of the myocardium during the AMI. It is currently believed that these cells help to regenerate the heart muscle and protect the left ventricle from unfavourable post-infarction remodelling [19]. This TRAIL activity related to MSC, together with the anti-inflammatory and anti-atherosclerotic effects of TRAIL, means that the regulation of its activity by tumour necrosis factor -alpha (TNF-α) and OPG continues to arouse interest. It is possible that the increase in OPG concentrations, which reduces the positive effects of TRAIL on MSCs and on the vascular system, may have important pathogenic implications for AMI [19]. The prospect of disabling this OPG action is an exciting concept in treating CVDs in the future.

Research is also underway to determine the possibility of diminishing the effects of OPG on TRAIL, where it acts on the vascular cell walls. Of particular interest are the studies which demonstrate that TRAIL stimulates endothelial nitric oxide synthase (eNOS) and increases the production of nitric oxide (NO) in ECs [48]. The results, to date, have been encouraging, as the search continues to eliminate the unwanted effects of OPG and enhance the effect of TRAIL in the vascular system. Whether this can be achieved or, indeed, whether it will prove to be beneficial is still unknown, but bearing in mind the mechanisms of action of OPG and TRAIL in the cardiovascular system, it does appear to be a potential therapeutic target in CVDs.

Furthermore, it is thought that OPG is a chemotactic factor in the infiltration of inflammatory cells into the vascular wall, which occurs in the initial stages of atherosclerotic lesions [4,25,49,50,51,52,53]. Pro-inflammatory cytokines, such as IL-6 and interleukin-1ß (IL-1ß), are sourced from activated inflammatory cells. The ability of OPG, firstly, to interact with these cytokines and, secondly, to enhance adhesion molecule expression in ECs now forms the rationale for the search for new strategies in the treatment of CVDs, with the cytokines and their receptors as potential therapeutic targets. 

This knowledge concerning the mechanisms of action of OPG on the vascular wall has led to a better understanding of the clinical observations, which demonstrate a strong relationship between CVDs and high OPG concentrations. These mechanisms of action are also now considered extremely important for the pathogenesis of CVDs and for future CVD therapies. Researchers are currently investigating what the best methods may be for neutralising the effects of OPG on the cells in the CV system and whether these methods will provide clinical benefits. There is no doubt, however, that, due to these mechanisms of action on the CV system, the signal axis with a central role of OPG is a potential therapeutic target in CVDs.

For the next step in our study, we plan to carry out in vivo research to assess the differences in responses to Ang II in OPG^+^ and OPG^−^ mice. This will be performed based on a murine model of endothelial dysfunction induced by long-term intraperitoneal administration of Ang II with an osmotic pump. This experimental model [54] will allow us not only to assess the endothelial function but also to compare vascular remodelling, hypertension, oxidative stress and damage to various organs, as well as cytokine and adhesion molecule expression in OPG^+^ and OPG^−^ mice exposed to Ang II. 

### 3.5. Limitation of the Study

The main limitation of this study is the use of the mouse model. Due to the complex regulation of OPG in the human body, a mouse model with the knockout of the OPG gene may not fully correlate to human physiology. Moreover, although Ang II is widely used in experiments as an inductor of endothelial dysfunction, it does not fully reflect the complexity of such dysfunction.

## 4. Materials and Methods

**Figure 5:**
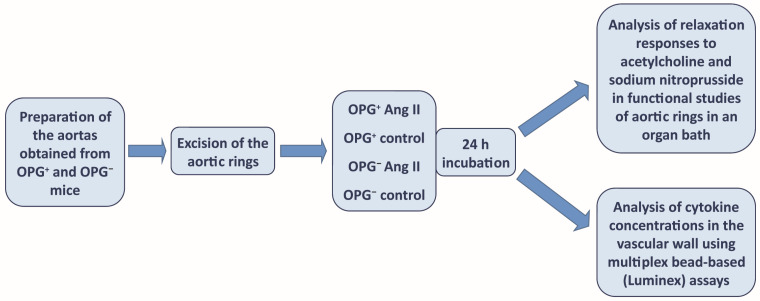
Research diagram.

Thoracic aortic segments from male wild-type mice (C57BL/6J, OPG^+^) and from OPG-knockout mice (B6.129.S4-OPG, OPG^−^), which were directly derived from C57BL/6J mice, were used for the experiment. The animals were purchased from The Jackson Laboratory, Farmington, CT, USA. All mice were kept under specific pathogen-free conditions (SPF) and fed a standard laboratory diet and water ad libitum. All experimental procedures used in this study were carried out in accordance with the Guidelines for the Care and Treatment of Animals of the European Communities and the Guide for the Care and Use of Laboratory Animals published by the US National Institutes of Health (NIH publication number 85-23, revised 1996). The mice were anaesthetised with ketamine/xylazine (100/10 mg/kg body weight). Each thoracic aorta was quickly removed and placed in Krebs-Heinseleit (KH) buffer with the following composition (mM): NaCl 118.0, CaCl_2_ 2.52, MgSO_4_ 1.16, NaHCO_3_ 24.88, KH_2_PO_4_ 1.18, KCl 4.7, glucose 10.0, pyruvic acid 2.0, EDTA 0.5. Aortas were carefully dissected from the surrounding tissues and cut into rings (2 mm long). Aortic rings were randomly divided into experimental groups (control, Ang II) and incubated for 24 h in medium (MEM, 0.1% FBS) only (control group) or with Ang II (1 µM) (Ang II group), at 37 °C (CellCulture CO_2_ incubator, ESCO), as previously described by other authors [31]. After 24 h of incubation, the aortic rings were directly transferred to the organ bath for functional studies using a set of myographs. In another part of the experiment, after the same 24 h of incubation, the aortic rings were frozen in PathScan Lysis Buffer (Cell Signaling Technology, Danvers, MA, USA) at −80 °C for subsequent biochemical analyses (Figure 5).

### 4.1. Evaluation of Nitric Oxide-Dependent Endothelial Function in the Isolated Murine Aorta

After the 24 h of incubation described above, the aortic rings were mounted on two pins in the organ chambers of the wire myograph (620 M, Danish Myo Technology, Hinnerup, Denmark), filled with 5 mL of KH buffer, and gassed with carbogen (95% O_2_, 5% CO_2_) at 37 °C. After assembly, the aortic rings were equilibrated for 30 min without stretching. The resting tension of the rings was then gradually increased to 10 mN. After the equilibration period, aortic viability was assessed by contractile responses to potassium chloride (30 and 60 mM of KCl), and maximal contraction to phenylephrine (Phe; 3 μM) was evoked. NO-dependent endothelial function was assessed by the relaxation response to cumulative concentrations of acetylcholine (ACh; 0.01–10 µM) in vessels pre-contracted with phenylephrine (Phe; 0.1–1 µM). Endothelium-independent relaxation was determined using cumulative concentrations of sodium nitroprusside (SNP; 0.001–10 µM). All tissue responses were recorded using a data-acquisition system and recording software (Power Lab, LabChart 3.01, AD Instruments, Bella Vista NSW, Australia). The relaxation response was expressed as a percentage of the pre-contraction induced by phenylephrine.

### 4.2. Fluorescent Bead-Based Luminex Cytokine Assay

The murine aortic rings, which had been previously frozen at −80 °C, were thawed and homogenised on ice in PathScan Lysis Buffer (Cell Signaling Technology, Danvers, MA, USA) using TissueRuptor (Qiagen, Germantown, MD, USA). The solution was then centrifuged (10 min. at 10,000× *g* 4 °C), and the total protein in the supernatant was evaluated using the BCA method (MERCK KGaA, Darmstadt, Germany). The protein concentration was normalised, and an equal amount of total protein was used in the Luminex assay. Cytokine concentrations were measured using multiplex bead-based (Luminex) assays on a Bio-Plex 200 Suspension Array System according to the manufacturer’s instructions. Bio-Plex Pro Mouse Assays (Bio-Rad Laboratories, Watford, UK) were used. Data were acquired on a validated and calibrated Bio-Plex 200 system (Bio-Rad Laboratories, Watford, UK) and analysed using Bio-Plex Manager 6.0 software (Bio-Rad Laboratories, Watford, UK) with a detection target of 50 beads per region, high RP1 target for CAL2 calibration, and recommended doublet discriminator (DD) gates of 5000–25,000 for Bio-Plex. The median fluorescence intensity (MFI) was measured. The concentration of each cytokine was calculated from the standard curve using Bio-Plex software 6.0 (Bio-Rad Laboratories, Watford, UK).

### 4.3. Statistical Analysis

Data were analysed by Prism 9.5.0 software (GraphPad, CA, USA). Results are presented as the mean ± SEM. The normality of the results was analysed using the Shapiro–Wilk test. To calculate statistical significance, an Unpaired Student’s *t*-test or Mann–Whitney test was used.

## Data Availability

The data that support the findings of this study are available from the corresponding author upon reasonable request.
